# Dietary Interventions in Obesity and Metabolic Syndrome

**DOI:** 10.3390/nu15112513

**Published:** 2023-05-29

**Authors:** Karolina Szewczyk-Golec, Iga Hołyńska-Iwan

**Affiliations:** 1 Department of Medical Biology and Biochemistry, Faculty of Medicine, Ludwik Rydygier Collegium Medicum in Bydgoszcz, Nicolaus Copernicus University in Toruń, 85-092 Bydgoszcz, Poland; 2 Department of Pathobiochemistry and Clinical Chemistry, Faculty of Pharmacy, Ludwik Rydygier Collegium Medicum in Bydgoszcz, Nicolaus Copernicus University in Toruń, 85-094 Bydgoszcz, Poland; igaholynska@cm.umk.pl

Here, we present a Special Issue of *Nutrients* entitled “Specialized Diet, Obesity and Associated Metabolic Diseases” containing up-to-date scientific data important to both researchers and the public. The Special Issue consists of four reviews and eight original articles, the results of which are important for understanding the mechanisms occurring in the course of obesity and metabolic syndrome. Moreover, the presented reviews are a reliable source of structured scientific knowledge in the field of dietary treatment of patients with obesity.

The European Union report from 2022 states that no less than 60% of European adults are overweight or obese [1]. Among children, 27% of girls and 29% of boys suffer from overweight/obesity. Due to the fact that obesity and metabolic syndrome are important factors that deteriorate the health of the population and participate in the development of non-communicable diseases, as well as worsen the prognosis and reduce the effectiveness of therapy for a number of diseases, the European Union and other countries have announced life-course obesity prevention strategies [1,2].

In this Special Issue, great emphasis was placed on the study of metabolic changes in the course of obesity and metabolic syndrome using the measurements of morphometric parameters, such as body composition, and the laboratory parameters of glucose and lipid metabolism, among others. Particularly noteworthy are studies involving children and adolescents, as an early fight against negative metabolic changes may prove to be an effective tool to improve health and allow for satisfactory personal development in this group of patients. Castillo-Valenzuela et al. [3] surveyed children aged 4–14 for obesity and micronutrient deficiencies. Deficiencies in vitamin D, calcium, copper, iron, and zinc were revealed in children from different cities in Chile. In addition, it was indicated that the low socioeconomic status of children and improper dietary habits correlated positively with the development of obesity, which was additionally associated with the presence of deficiencies of micronutrients, especially vitamin D. In the article of Dettlaff-Dunkowska et al. [4], the effects of implementing the “6-10-14 for Health” program in 152 children aged 6–15 with a BMI exceeding the 85th percentile were described. Body composition parameters, such as fat mass and free-fat mass, were measured using two methods. The results were not surprising, as it was confirmed that a balanced diet and physical activity had a positive effect on the body composition, reducing fat mass and increasing fat-free mass. For people working with children, it seems particularly important to prove that, regardless of the measurement method chosen, the effects of reducing body fat in children and adolescents can be reliably assessed.

To fight the obesity epidemic, various therapeutic strategies using specialized diets and/or supplementation with various bioactive substances are being evaluated in a number of studies. In a randomized controlled trial, patients with obesity and metabolic syndrome were randomly divided into four groups that differed in the intensity of physical activity and supplementation with arginine and leucine [5]. Body composition, metabolic syndrome and cardiorespiratory parameters, as well as markers of inflammation, glucose and lipid metabolism, were analyzed. It was shown that both a 6-month arginine and leucine supplementation and supervised adapted physical activity failed to improve the parameters of metabolic syndrome in obese patients. However, physical training improved the cardiorespiratory parameters.

Dietary interventions that affect changes in laboratory parameters in obesity and/or metabolic syndrome that may have an impact on arterial stiffness were summarized in a review by Stanek et al. [6]. Arterial stiffness, as a new predictor of cardiovascular risk, may be modified by dietary habits. The review considered the determinants of the Western diet, such as high intakes of sodium, fatty acids and cholesterol, while low intakes of vegetables and potassium. In easy-to-read tables, data on the Western diet were compared with the Mediterranean diet. By organizing the scientific evidence in the review, it can be seen that dietary recommendations to prevent arterial stiffness should be similar to the Mediterranean diet, including high consumption of dairy products, vegetable oils and fish; five servings of fruit and vegetables a day; and avoiding red meat.

In obesity research, animal model studies are especially important to assess the impact of various dietary interventions under strictly controlled experimental conditions. Rodrigez-Perez et al. [7] reported the results of their research on the effect of extra virgin olive oil on inhibiting the development of diabetic nephropathy in an animal model. The administration of 3′,4′-dihydroxyphenylglycol, a polyphenol from olive oil, to rats with diabetic kidney disease normalized urinary protein excretion and renal parameters (creatinine clearance), as well as the reduced markers of oxidative stress. The normalization of oxidative stress parameters and liver tests, as well as weight loss, was also achieved by using a restrictive ketogenic diet in Wistar rats [8]. There were no changes in the advanced glycation end-products and hematological parameters. The authors suggested that following a ketogenic diet may be particularly beneficial for improving liver function. Interestingly, it was shown that duodenojejunal omega switch surgery, used in the treatment of extreme obesity, can be effective even when patients do not follow the recommended diet [9]. In the experiment, the use of a cafeteria diet (high fat-sugar diet) in Sprague Dawley rats subjected to the above operation had a negative impact on the parameters of oxidative stress but did not completely destroy the effects of the bariatric treatment.

Particularly noteworthy is the use of natural products in the fight against obesity and metabolic syndrome. Citizens of modern societies are looking for ecological products that contain substances of plant origin and, additionally, do not burden the environment. In this Special Issue, a description of the results of research on these concerns can be also found. Kim and Jeong [10] compiled data showing that plant-derived phytochemicals can normalize the number of ceramides and potentially harmful sphingolipids. The dysregulation of the production and the activity of ceramides is of particular importance for both the development of obesity and the effectiveness of its treatment. The authors systematically reviewed the results of clinical, animal and in vitro studies on this topic. Kang et al. [11] showed that ginsenoside has an anti-inflammatory effect by inhibiting macrophage activity and increasing energy consumption, which regulates cellular metabolism in terms of glycolysis and mitochondrial respiration. In turn, Gackowski et al. [12] in their in silico study proved the potential positive effect of stevioside, a natural sweetener, on insulin resistance. It has been confirmed that the use of stevioside may have a high probability of inhibiting progressive insulin resistance due to the fact that it blocks activated coagulation factor X. The possibility of creating new isosteviol analogues with enhanced pharmacological activity was also demonstrated.

Changes in the intestinal microbiome have been recognized for years as contributing to the development of metabolic and oncological diseases, and the restoration of the natural microflora in humans is a factor supporting convalescence. Bagheri et al. [13] organized the data on the obesity-related gut microbiota and the impact of inflammation on changes in this area. This review presents a significant amount of scientific evidence that the use of an anti-inflammatory diet regulates inflammation and the microbiome, which has a particularly positive effect on the inhibition of adipocyte activity. In turn, Park et al. [14] focused on the results of research in the field of postbiotics in diet-induced metabolic syndrome. Postbiotics, substances produced or released by micro-organism metabolic activities, play an important role in maintaining the physiological microbiota and thus the good condition and health of people. The authors placed particular emphasis on the use of postbiotics as potential therapeutic agents used in anti-diabetic, anti-obesity and anti-hypertensive therapies.

Due to the importance of the problem, the dissemination of knowledge about the mechanisms related to the development, treatment and prevention of obesity and metabolic syndrome is valuable for physicians on the frontline of the fight against these diseases [2]. However, the presented articles may also be of interest to dieticians, physiotherapists, trainers and teachers who want to broaden their knowledge in the field of pathophysiological changes occurring in the course of obesity, as well as the possibilities of its treatment, and above all, for all those interested in the prevention and treatment of lifestyle diseases.
